# Mortality rate of pulmonary infection in senile dementia patients: A systematic review and meta-analysis

**DOI:** 10.1097/MD.0000000000039816

**Published:** 2024-09-20

**Authors:** Jianning Yao, Shunlin Liu, Qun Chen

**Affiliations:** a Department of Psychiatry, Huzhou 3rd Hospital, Huzhou, Zhejiang Province, China; b Department of Respiratory Medicine, Huzhou Hospital of Zhejiang University, Huzhou, Zhejiang Province, China.

**Keywords:** dementia, mortality rate, old age, pulmonary infection

## Abstract

**Background:**

Dementia is estimated that this kind of neurodegenerative disease directly affects 50 million patients worldwide. About 12% to 70% death of dementia disease can be attributed to pneumonia. We aimed to evaluate the pneumonia-related mortality of dementia patients and how the frequency of pneumonia-related death varies according to the data of death (autopsy or death certificate).

**Methods:**

English literatures published from PubMed and Embase databases were extracted. Stata/SE 16.0 software was used for statistical analysis.

**Results:**

In the end, a total of 7 studies were finally included in this meta-analysis. The results showed that: (1) The total mortality rate associated with pneumonia was 24.68% (95% confidence interval [CI]: 19.07%, 30.29%); (2) The pneumonia-related mortality rate of dementia patients confirmed by autopsy was 56.14% (95% CI: 32.36%, 79.92%); (3) The pneumonia-related mortality rate of dementia patients confirmed by death certificate was 16.12% (95% CI: 9.98%, 22.26%); (4) The pneumonia-related direct mortality rate of dementia patients was 50.07% (95% CI: 34.85%, 65.30%); (5) The pneumonia-related indirect mortality rate of dementia patients was 12.43% (95% CI: 5.85%, 19.00%); (6) The hospital-reported mortality rate of dementia patients related to pneumonia was 12.66% (95% CI: 6.60%, 18.72%); (7) The mortality rate of dementia patients related to pneumonia was 17.48% (95% CI: 10.60%, 24.38%).

**Conclusion:**

This meta-analysis shows that the pneumonia-related mortality of dementia patients is much higher than the expectation of clinicians. The results of the study greatly warned clinicians to pay close attention to pneumonia cases of senile dementia patients.

## 1. Introduction

Dementia is the most common type of neurodegenerative disease accounting for 60% to 80% of cases. It is estimated that this kind of neurodegenerative disease directly affects 50 million patients worldwide.^[1]^ Dementia patients usually show obvious amnesia cognitive impairment, and a few show non-amnesia cognitive impairment. Among them, dementia patients with short-term memory impairment are the most common. In addition to memory impairment, dementia patients also have certain obstacles in language expression, visual processing, and work execution. A previous study on 184 patients with dementia showed that 31% of them only had pathological changes of dementia, 22% had pathological changes of dementia plus α-synuclein, 29.5% had pathological changes of dementia plus TAR DNA binding protein-43, and 17.5% had pathological changes of dementia plus α-synuclein and TAR DNA binding protein-43.^[2]^ In 2019, a survey showed that the number of patients with dementia is expected to increase to 131 million in 2050 worldwide.^[3]^ Subsequently, another study in 2017 showed that the incidence of dementia decreased slightly in some high-income countries such as Britain, the United States, and France, but it increased steadily in low- and middle-income countries.^[4]^

Studies have shown that a considerable part of the death of dementia can be attributed to pneumonia.^[5]^ The daily severe dysphagia, low activity, and bedridden of dementia patients are all potential reasons for the high incidence of pneumonia. At present, the risk factors of pneumonia in dementia patients are not clear, and some researchers suggest that it may be because dementia patients are prone to dysphagia, which leads to inhalation of oral contents into the respiratory tract, thus leading to aspiration pneumonia.^[6]^ Some researchers believe that the immune deficiency of dementia patients may lead to the development of pneumonia.^[7]^ Previously, a meta-analysis showed that the probability of pneumonia-related death in patients with dementia was more than twice as high as that in patients without dementia.^[8]^ However, it is reported that the death frequency related to pneumonia varies among the elderly with dementia, ranging from 12% to 70%.^[9–14]^

The purpose of this study is to clarify the pneumonia-related mortality rate of dementia patients and how the frequency of pneumonia-related death varies according to the data of cause of death (autopsy or death certificate). The results of this study are helpful for the clinical management of preventing pneumonia in dementia patients and maximizing the life expectancy of these patients.

## 2. Method

### 2.1. Literature retrieval strategy

This meta-analysis is carried out in strict accordance with the Preferred Reporting Items for Systematic Reviews and Meta-Analyses statement. In this study, English literatures published from PubMed and Embase databases. The specific retrieval date is from the establishment of the database to September 30th, 2023. The English retrieval formula of literature retrieval is: “(Dementia or Alzheimer’s Dementia or Alzheimer’s disease or Dementia with Lewy bodies or different Lewy body disease or” vascular dementia OR frontotemporal dementia OR mixed-type of dementia) AND (pneumonia OR lower respiratory tract infection OR bronchopneumonia OR aspiration pneumonia OR nosocomial pneumonia OR community-acquired pneumoni a OR nursing and healthcare-associated pneumonia OR ventilator-associated pneumonia) AND (mortality OR death OR comorbidity).” Two authors searched studies according to the search strategy, independently. First, duplicated studies were excluded. Then, titles and abstracts were scanned to find possible studies. Finally, the full text would be read to filter for eligible studies with sufficient data. When there was a dispute on the inclusion of a study, the third author would decide on it.

### 2.2. Inclusion criteria and exclusion criteria

Inclusion criteria:

1. Study design: randomized controlled trial, cohort study, and cross-sectional study;
2. Population: elderly people over 70 years old with or without dementia (as control group);
3. Observation index: Death of pneumonia related to dementia;
4. Main results: Distribution of pneumonia-related deaths.

Exclusion criteria:

1. repeated articles or no full text;
2. Only the approximate frequency of pneumonia-related deaths was provided, but there was no exact number of patients;
3. Research on data missing or errors that cannot be completed and corrected;
4. Lack of outcome indicators needed in this study;
5. Letters, case reports, comments, practice guides, etc;
6. Patients with other basic diseases are included;
7. All animal experiments.

### 2.3. Data extraction

We read the full text of the included studies and collected the reported mortality rate of pulmonary infection in senile dementia patients in tables, figures or results section. The 2 authors of this study independently extracted the data included in the study into standardized tables, and resolved any differences through consensus or participation of the third author. We collected information about bias risk, characteristics of participants, characteristics of intervention group and control group, and results. If we cannot calculate the effect, we contacted the author of the experiment and ask for more data.

### 2.4. Definitions

Pneumonia-related mortality confirmed by autopsy was defined as data obtained from autopsy reports and clinical information as available from the medical records. Pneumonia-related mortality confirmed by certification was defined as data obtained from death certificates and clinical information as available from the medical records, that did not require an autopsy on the patients. Pneumonia-related direct mortality was defined as deaths directly attributable to pneumonia. Pneumonia indirect mortality was defined as pneumonia combined with 1 or more other causes of death, which were difficult to prioritize and which combine to cause death in the same case. Hospital-reported mortality associated with pneumonia was defined as pneumonia-related deaths reported from hospital. Pneumonia-related socially reported mortality was defined as pneumonia-related deaths reported from community hospitals or government offices.

### 2.5. Quality evaluation

Two independent researchers evaluated the quality of literature based on Newcastle-Ottawa Scale (NOS Scale) 11, and reached an agreement with a third party in case of disagreement. NOS scale includes 3 dimensions, including 8 items: 4 items for the selection of research objects (representative of experimental group, representative of control group, definition of experimental group, and definition of control group), 1 item for the comparability between groups and 3 items for the measurement of results (outcome index measurement, follow-up time, and follow-up integrity); except for the comparability between groups, the highest score is 2, and the highest score for other items is 1, with a score range of 0 to 9. The higher the total score, the higher the research quality. It is considered that the score of 6 to 9 is high-quality research, and the score of 0 to 5 is low-quality.

### 2.6. Statistical methods

In this study, Stata/SE 16.0 software is used for data analysis. In the whole meta-analysis, we used random effect model and general inverse variance method to calculate pneumonia-related mortality, and the confidence interval (CI) was 95%. Q test is used to test the heterogeneity between studies. If *I*^2^ is <50% and *P* < .1, it means that the heterogeneity between studies is small, so the fixed effect model is used. Otherwise, the random effect model is used. This paper describes the statistical results of meta-analysis with forest map.

## 3. Results

### 3.1. literature search screening results

**Figure 1.**
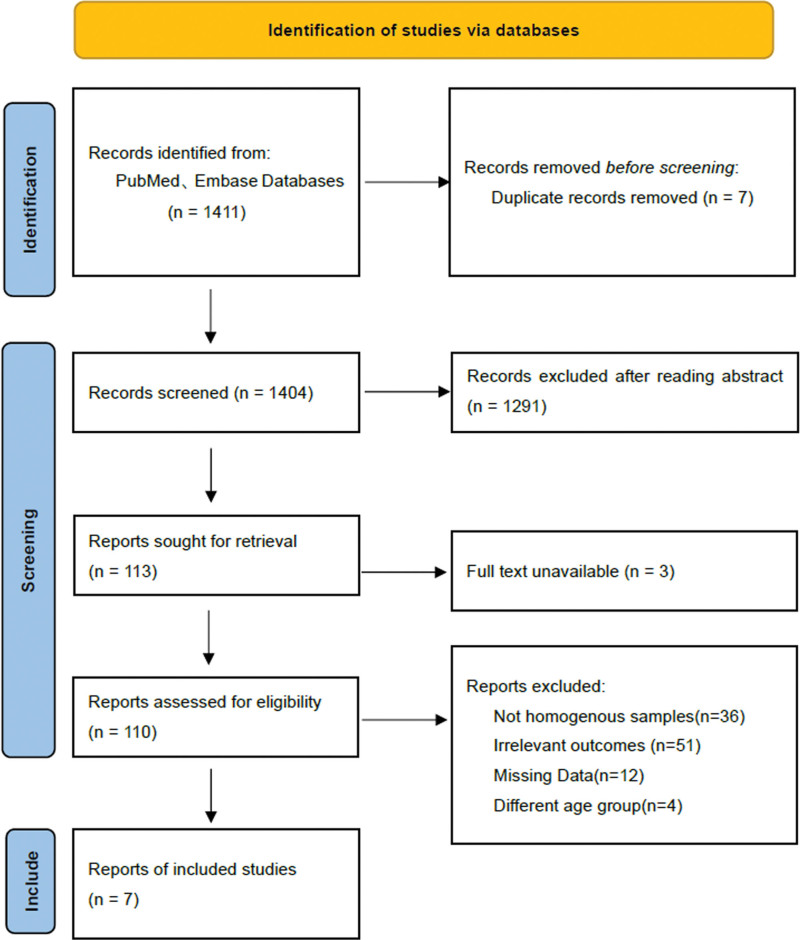
Flow chart of document screening.

Through the above retrieval method, a total of 1411 studies were extracted from 2 databases, and 1404 original studies were extracted after eliminating duplicate studies. By browsing the titles, keywords and abstracts, it is determined that 113 studies may be related to the research topic. We further searched the full texts of these studies and got the full texts of 110 studies. According to the inclusion and exclusion criteria, we excluded another 101 studies. Finally, a total of 7 studies were finally included in this meta-analysis, as shown in Figure 1.

### 3.2. Basic characteristics of inclusion in the study

All the 7 included studies are original studies. The basic characteristics of the study are shown in Table 1.

**Table 1:** Characteristics of included literature.

Author, year	Research location	Research type	Sample size	Average age ± standard deviation	Female (%)
van de Vorst 2016^[15]^	Netherlands	Perspective study	39,164	81.4 ± 7.0	61.3
Manabe 2015^[16]^	Japan	Retrospective study	157	84.5 ± 8.5	62.4
Tschanz 2004^[11]^	Sweden	Perspective study	355	83.3 ± 7.0	64
Laditika 2005^[17]^	America	Retrospective study	36,887	85.0 ± 6.9	54
Ganguli 2005^[18]^	America	Perspective study	1670	73.4 ± 5.9	57.8
Todd 2013^[19]^	America	Perspective study	396	78.6 ± 7.5	68.3
Magaki 2014^[10]^	America	Retrospective study	86	78.5 ± 11.5	47.7

### 3.3. Quality evaluation of included documents

Evaluation of literature quality based on NOS scale, with scores ranging from 0 to 9. It is considered that the scores of 6 to 9 are high-quality studies, and the scores of 0 to 5 are low-quality studies. The quality evaluation results of the included literatures are shown in Table 2.

**Table 2:** Quality evaluation table for inclusion in the study.

Inclusion study	Quality evaluation score (NOS)	Selection of research objects				Comparability between groups	Measurement of outcome		
		Representative of experimental group	Representative of control group	Definition of experimental group	Definition of control group		Outcome index measurement	Follow-up time	Follow-up integrity
van de Vorst 2016^[15]^	9	☆	☆	☆	☆	☆☆	☆	☆	☆
Manabe 2015^[16]^	8	–	☆	☆	☆	☆☆	☆	☆	☆
Tschanz 2004^[11]^	9	☆	☆	☆	☆	☆☆	☆	☆	☆
Laditika 2005^[17]^	6	–	–	☆	☆	☆	☆	☆	☆
Ganguli 2005^[18]^	8	–	☆	☆	☆	☆☆	☆	☆	☆
Todd 2013^[19]^	7	☆	☆	☆	☆	☆☆	–	☆	–
Magaki 2014^[10]^	9	☆	☆	☆	☆	☆☆	☆	☆	☆

### 3.4. Meta-analysis results

#### 3.4.1. Total mortality associated with pneumonia in dementia patients

**Figure 2.**
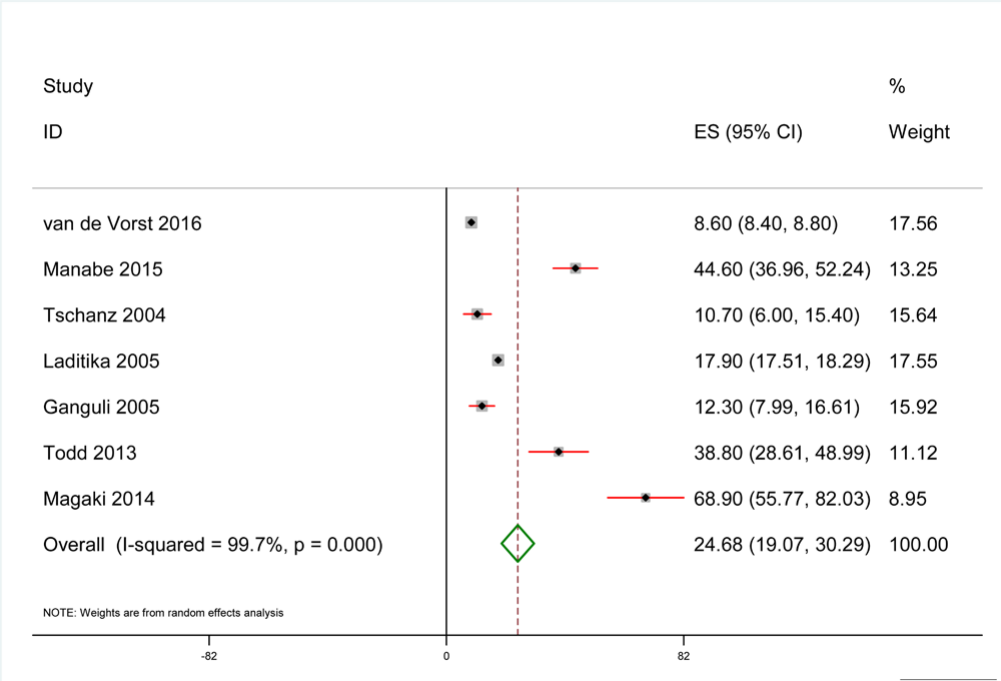
Total mortality associated with pneumonia in dementia patients.

The results showed that, as shown in Figure 2, the mortality rate associated with pneumonia in dementia patients was 24.68% (95% CI: 19.07%, 30.29%). The results of heterogeneity test included in the study were: *I*^2^ = 99.95%, *P* < .001.

#### 3.4.2. Pneumonia-related mortality of dementia patients confirmed by autopsy

**Figure 3.**
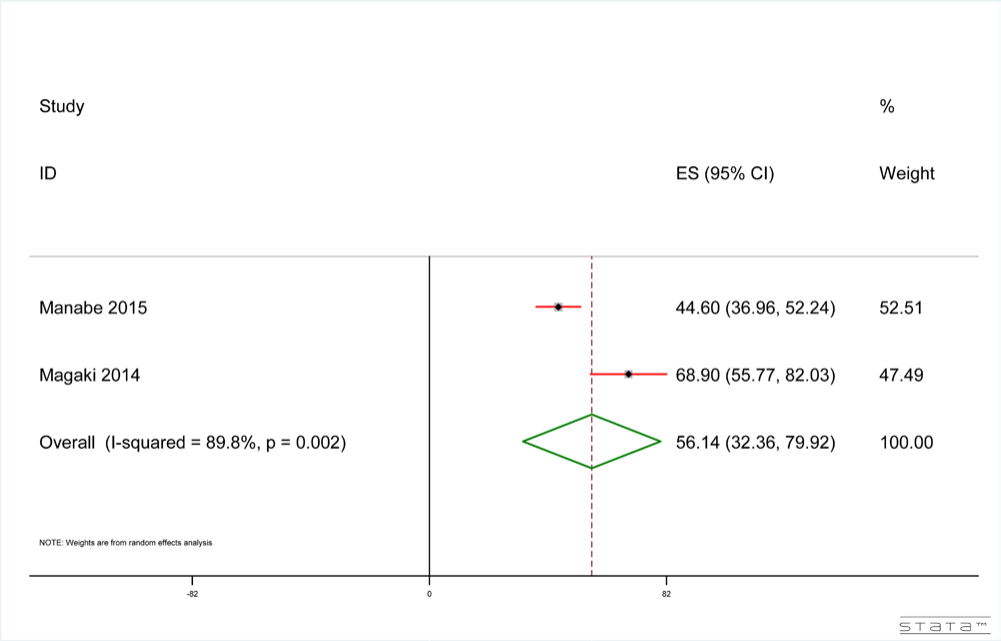
Forest plot of pneumonia-related mortality confirmed by autopsy in dementia patients.

The results showed that, as shown in Figure 3, the pneumonia-related mortality rate of dementia patients was 56.14% (95% CI: 32.36%, 79.92%). The result of heterogeneity test included in the study is: *I*^2^ = 89.83%, *P* < .001.

#### 3.4.3. Pneumonia-related mortality of dementia patients confirmed by death certificate

**Figure 4.**
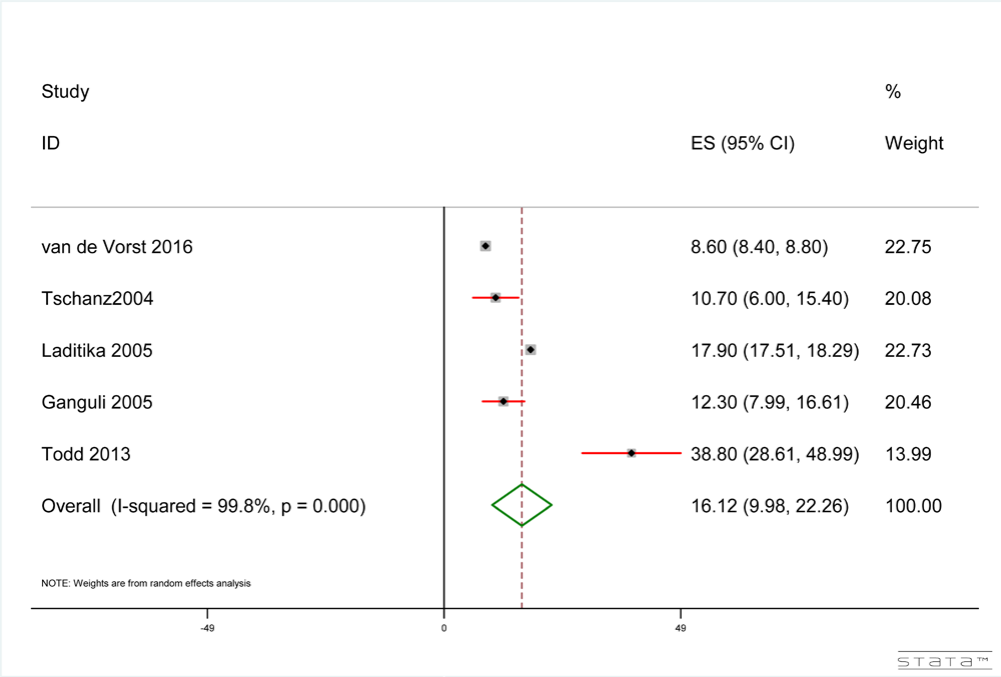
Forest plot of pneumonia-related mortality confirmed by death certificate in dementia patients.

The results showed that, as shown in Figure 4, the pneumonia-related mortality rate of dementia patients confirmed by death certificate was 16.12% (95% CI: 9.98%, 22.26%). The results of heterogeneity test included in the study were: *I*^2^ = 99.80%, *P* < .001.

#### 3.4.4. Pneumonia-related direct mortality of dementia patients

**Figure 5.**
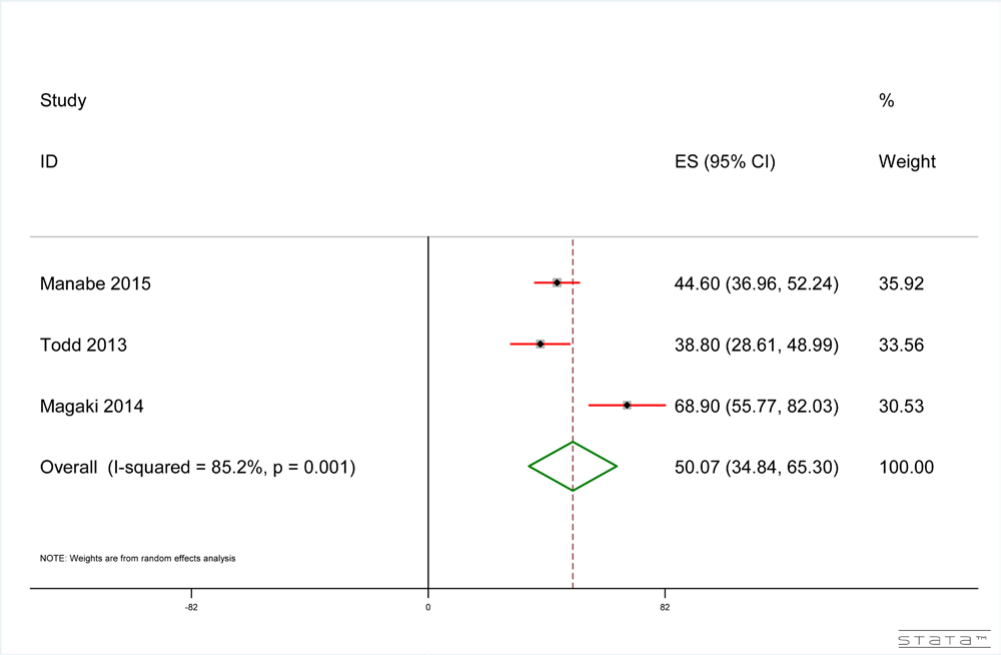
Forest plot of pneumonia-related direct mortality in dementia patients.

The results showed that, as shown in Figure 5, the direct mortality rate of dementia patients related to pneumonia was 50.07% (95% CI: 34.85%, 65.30%). The result of heterogeneity test included in the study is: *I*^2^ = 85.2%, *P* < .001.

#### 3.4.5. Pneumonia-related indirect mortality of dementia patients

**Figure 6.**
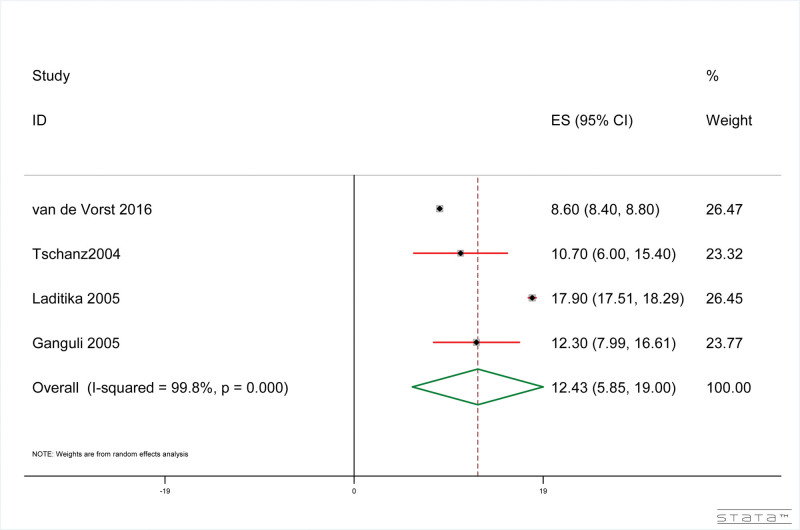
Forest plot of pneumonia-related indirect mortality in dementia patients.

The results showed that, as shown in Figure 6, the pneumonia-related indirect mortality rate of dementia patients was 12.43% (95% CI: 5.85%, 19.00%). The results of heterogeneity test included in the study were: *I*^2^ = 99.8%, *P* < .001.

#### 3.4.6. Hospital-reported mortality of dementia patients with pneumonia

**Figure 7.**
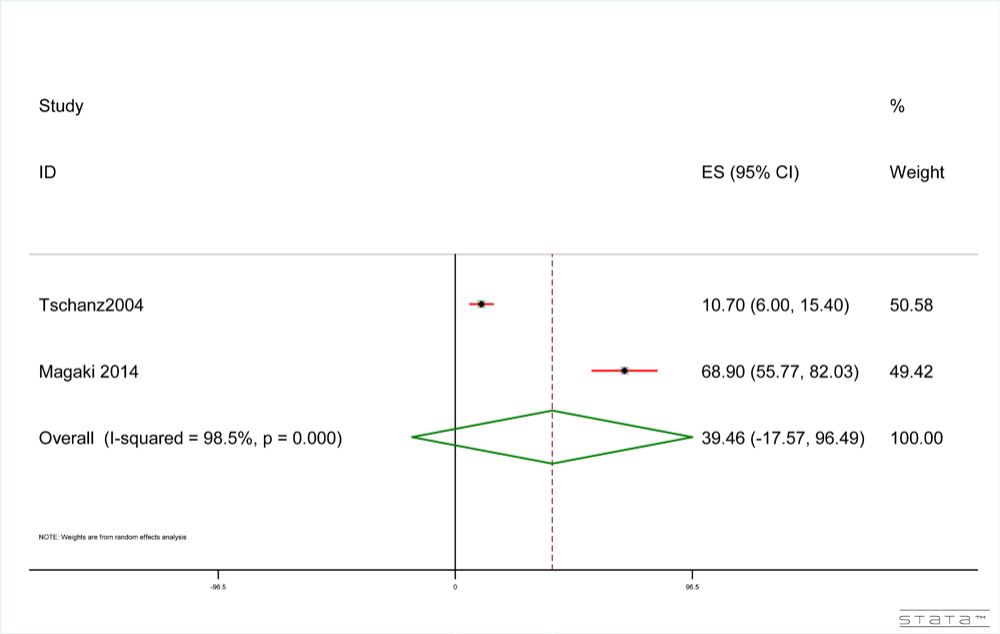
Hospital-reported mortality associated with pneumonia in dementia patients.

The results showed that, as shown in Figure 7, the hospital-reported mortality rate of dementia patients was 39.46% (95% CI: ‐17.57%, 96.50%). The results of heterogeneity test included in the study were: *I*^2^ = 98.51%, *P* < .001.

#### 3.4.7. Dementia patients with pneumonia-related social reported mortality

**Figure 8.**
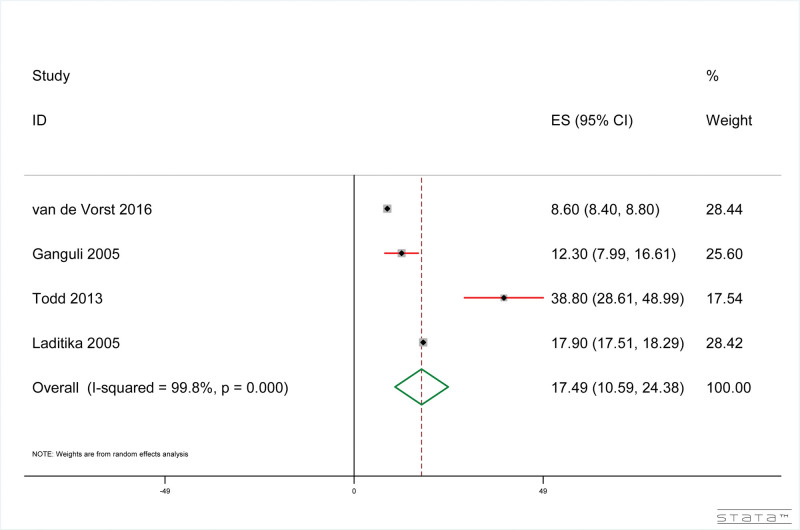
Pneumonia-related socially reported mortality in dementia patients.

The results showed that, as shown in Figure 8, the mortality rate of dementia patients related to pneumonia was 17.48% (95% CI: 10.60%, 24.38%). The results of heterogeneity test included in the study were: *I*^2^ = 99.8%, *P* < .001.

#### 3.4.8. Publication bias

**Figure 9.**
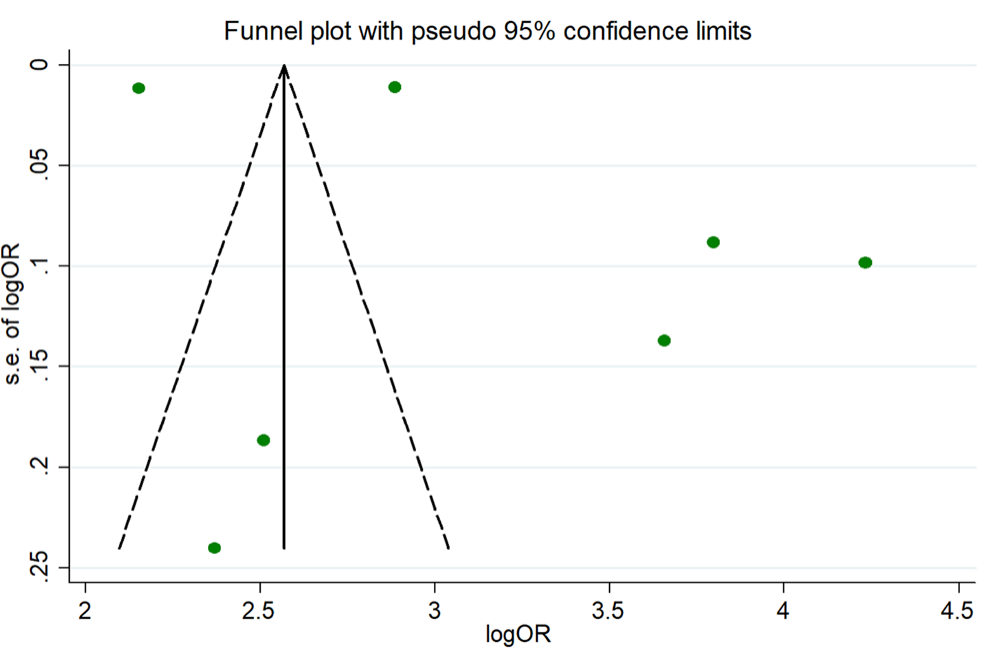
Funnel plot.

The funnel diagram shows that there is a slight asymmetry in the inclusion study (Fig. 9), and we speculate that there may be some publication bias. Further, we use Egger method to evaluate the publication bias, and the results show that there is no publication bias (Egger test, *t* = 0.71, *P* = .51).

## 4. Discussion

This meta-analysis and systematic review finally included 7 studies and found that 24.68% (95% CI: 19.07%, 30.29%) of dementia patients died from pneumonia. Among them, there was a higher mortality rate reported in hospitals (12.66% [95% CI: 6.60%, 18.72%]) compared to the pneumonia-related dementia mortality rate reported in society (17.48% [95% CI: 10.60–24.38%]).

Dementia is a chronic neurodegenerative disease, that is the 6th leading cause of death in the United States. At present, there is no effective treatment, and its progress can only be delayed through disease rehabilitation.^[20,21]^ According to the report of the International Alzheimer’s Disease Organization in 2018, there are about 50 million people with dementia worldwide, which is expected to triple by 2050, and about two-thirds of them live in low-income and middle-income countries.^[22]^ A systematic review concluded that pneumonia, cardiovascular disease, and Alzheimer disease or related dementias are the most common causes of death in people with dementia.^[23]^ However, the drugs for treating dementia are limited. In order to help patients live longer, it is urgent to develop the clinical treatment strategies for dementia. Therefore, it is essential to fully understand the impact of pneumonia on the mortality of dementia patients and to develop targeted strategies to improve the prognosis of dementia patients.

Previously, some studies have reported the pneumonia-related death on dementia patients. In 2019, Corona Virus Disease 2019 swept the world. A cohort study in Britain showed that dementia patients were more likely to get pneumonia than the control group (OR = 1.35, 95% CI: 1.23–1.48),^[24]^ which further confirmed the results of this study. At the same time, from December 2020 to August 2021 in the United States, a retrospective survey of the elderly who had received Corona Virus Disease 2019 showed that compared with those who had not received the vaccine, the risk of lung infection in patients with dementia increased significantly.^[25]^ In addition, a survey data from Korea National Health Database shows that compared with the control group, dementia patients are more prone to lung infection (OR = 2.11, 95% CI: 1.79–2.50).^[26]^ Therefore, this meta-analysis and systematic review aimed to comprehensively understand the role of pneumonia in dementia patients’ deaths by including multiple studies to expand the sample size.

Currently, the mechanisms underlying pneumonia-related death of dementia patients are not clear. In addition to the influence of age, complications and genetic factors, immune and inflammatory reactions also play an important role in increasing the death risk of dementia patients. In 2020, Mao et al showed that pneumonia can induce cytokine storms (including Interleukin-6, Interleukin-1β, and tumor necrosis factor) in human body, thus increasing the death risk of dementia patients.^[27]^ Subsequently, in 2021, Luigi Chiricosta and others proposed that due to inflammation, the levels of β-amyloid and neurotoxicity in human body increased, and pulmonary infection could lead to the increase of oxidative stress in human body, which would lead to the deterioration of dementia patients’ condition and increase the risk of death.^[28]^ The above mechanisms further explained that pneumonia, as reported in this study, was a significant cause of death in a considerable portion of dementia patients. This suggested that clinicians should not only pay attention to pneumonia-related symptoms in dementia patients but also closely monitor inflammatory markers and intervene actively.

For the higher pneumonia-related dementia mortality rate reported in hospitals compared to that reported in society, we speculate that this may be related to nosocomial infections resulting from prolonged hospitalization of dementia patients. Therefore, we suggest that dementia patients should minimize unnecessary hospitalizations, and clinics should strictly prevent nosocomial infections in dementia patients and avoid unnecessary antibiotic use. Meanwhile, the pneumonia-related dementia mortality rate reported in society was also noteworthy. We suggested that dementia patients might consider receiving regular influenza vaccinations to reduce the risk of pneumonia infection. In addition, family members of dementia patients should pay close attention to whether the patients were experiencing symptoms related to pneumonia.

This study provided a comprehensive evaluation of pneumonia-related deaths in dementia patients. However, further research is needed on measures to reduce pneumonia-related deaths in dementia patients, in order to explore more appropriate management strategies for dementia patients.

## 5. Conclusion

The pneumonia-related mortality of dementia patients is much higher than the expectation of clinicians, that greatly warned clinicians to take prompt action on pneumonia cases of senile dementia patients.

## Author contributions

**Conceptualization:** Jianning Yao.

**Data curation:** Jianning Yao, Shunlin Liu, Qun Chen.

**Formal analysis:** Qun Chen.

**Project administration:** Shunlin Liu.

**Writing – original draft:** Jianning Yao, Shunlin Liu, Qun Chen.

**Writing – review & editing:** Jianning Yao, Shunlin Liu, Qun Chen.
